# A modified U-Net to detect real sperms in videos of human sperm cell

**DOI:** 10.3389/frai.2024.1376546

**Published:** 2024-09-09

**Authors:** Hanan Saadat, Mohammad Mehdi Sepehri, Mahdi-Reza Borna, Behnam Maleki

**Affiliations:** ^1^Faculty of Industrial Engineering and Systems, Tarbiat Modares University, Tehran, Iran; ^2^Department of IT Engineering, Faculty of Industrial and Systems Engineering, Tarbiat Modares University, Tehran, Iran; ^3^Research and Clinical Center for Infertility, Yazd Reproductive Sciences Institute, Shahid Sadoughi University of Medical Sciences, Yazd, Iran

**Keywords:** male infertility 36–44, sperm segmentation, semen analysis (SA), deep learning—artificial intelligence, image segmentation—deep learning, U-Net

## Abstract

**Background:**

This study delves into the crucial domain of sperm segmentation, a pivotal component of male infertility diagnosis. It explores the efficacy of diverse architectural configurations coupled with various encoders, leveraging frames from the VISEM dataset for evaluation.

**Methods:**

The pursuit of automated sperm segmentation led to the examination of multiple deep learning architectures, each paired with distinct encoders. Extensive experimentation was conducted on the VISEM dataset to assess their performance.

**Results:**

Our study evaluated various deep learning architectures with different encoders for sperm segmentation using the VISEM dataset. While each model configuration exhibited distinct strengths and weaknesses, UNet++ with ResNet34 emerged as a top-performing model, demonstrating exceptional accuracy in distinguishing sperm cells from non-sperm cells. However, challenges persist in accurately identifying closely adjacent sperm cells. These findings provide valuable insights for improving automated sperm segmentation in male infertility diagnosis.

**Discussion:**

The study underscores the significance of selecting appropriate model combinations based on specific diagnostic requirements. It also highlights the challenges related to distinguishing closely adjacent sperm cells.

**Conclusion:**

This research advances the field of automated sperm segmentation for male infertility diagnosis, showcasing the potential of deep learning techniques. Future work should aim to enhance accuracy in scenarios involving close proximity between sperm cells, ultimately improving clinical sperm analysis.

## 1 Introduction

Infertility is a condition characterized by failing to achieve a clinical pregnancy after 12 months of regular and unprotected intercourse. It is a global problem, and around 15% of couples face it. Although male infertility is not well reported in many countries, research has shown that males are responsible for 20–30% of infertility, but 50% of cases are due to a combination of male and female factors.

The analysis of fertility factors is critical for diagnosis of male infertility and the treatment of patients. Commonly a specialist analyzes the sperms via a microscope. This method is complex and leans heavily on the skills of the expert, which may lead to observer variability (Popović and Thomas, 2017).

One of the systems used for semen analysis is Computer-aided sperm analysis (CASA). It was developed to add automation to the procedure in order to remove human factors. This system brings accuracy in analyzing different aspects of sperms. But there are some limitations to using this system.

First of all, the accuracy of the outcome can be influenced by many different factors. These factors are related to frame rate, sperm concertation, etc. (Lu et al., 2014). So, the outcome is so sensitive to different indicators like differences in semen biochemistry and the low quality of the image (Tomlinson and Naeem, 2018). Also, implanting CASA systems in laboratories is costly and comes with complexity.

Recent advancements in machine learning and artificial intelligence (AI) have shown promise in addressing these limitations. Deep learning, a branch of machine learning, emulates the way humans acquire knowledge and has been embraced for many imaging applications in healthcare, such as disease classification, edge detection, and image segmentation. The application of deep learning techniques to sperm analysis aims to enhance accuracy and reliability while reducing the dependency on highly skilled specialists.

Our study seeks to leverage these advancements by developing a modified U-Net architecture specifically tailored for sperm segmentation in microscopic images. The U-Net architecture is chosen for its proven efficacy in medical image segmentation, which is critical in accurately identifying and analyzing sperm cells amidst complex backgrounds. By incorporating advanced data augmentation techniques and transfer learning, we aim to improve the model’s performance in real-world conditions, addressing challenges such as closely adjacent sperm cells and varying image quality.

The primary objectives of this research are to enhance the accuracy of automated sperm segmentation and to provide a robust, reliable tool for clinical use in diagnosing male infertility. By improving the precision and efficiency of sperm analysis, we hope to contribute significantly to the field of reproductive health, ultimately aiding in better diagnosis and treatment outcomes for affected couples.

## 2 Related works

Automatic human sperm segmentation is a critical task and a variety of methods have been proposed in recent years. These methods were mostly based on thresholding, clustering, combining multiple color spaces, and convolutional neural networks (CNN).

Combining Otsu thresholding and wavelet transforms is one method for detecting sperm (Gonzalez-Castro et al., 2009; García-Olalla et al., 2015). Discrete Wavelet Transform (DWT) is a technique that describes texture patterns. This approach correctly segments all images that reach the description phase. In fact, this strategy rejects all the pictures that were not segmented correctly. Although some of the truly segmented images might be missed by this method (García-Olalla et al., 2015). Otsu thresholding is a simple, unsupervised method that can be used for image segmentation (Otsu, 1979).

The proposed technique (Ghasemian et al., 2015) for sperm region detection included different stages. The first stage of this algorithm contained the Sobel edge detection. In the next phase, they removed possible divergence in detected edges. After filling image holes and removing noises and small objects, only sperms remained in the output images.

Nissen et al. (2017) trained different deep convolutional neural network architectures in order to compare their capability for sperm cell segmentation and object detection. They used a dataset of 765 grayscale images and developed seven different architectures. They were not able to use many pooling layers in their networks due to the limitations related to computation time.

Marín and Chang (2021) proposed and compared two well-known CNN architectures for sperm segmentation task. They used the SCIAN-SpermSegG dataset to train Unet and Mask-RCNN on it. The size of the dataset was limited. So, they performed data augmentation. They also trained networks under two scenarios. No pre-trained weights were applied to the original architecture in the first scenario. However, in the second one, the influence of transfer learning was tested on each model.

Dewan et al. (2018) developed a method based on a blob detection technique (bankman 2002) for choosing potential sperm cells. By using Gaussian blur-based de-noising, they convert frames to grayscale images. In the next stage, they employ Sobel-based edge detection for extracting an edge image from the denoised image. The outcome will be a binary image by thresholding and removing noise in the final step.

Hicks et al. (2019) performed a multimodal analysis for predicting sperm motility. For the sperm detection part, they used Harris and Stephens corner detection algorithm (Harris and Stephens, 1988).

A recent study introduces a novel approach for estimating sperm count through computer-aided techniques, aiming to reduce the subjectivity in semen analysis. Utilizing object detection methods, this research focuses on estimating the motility and count of active sperm, tested on the Visem dataset. This approach underscores the potential of computer-aided methods in enhancing the accuracy of semen analysis, reporting a not-super tuned result with a mean Average Precision (mAP) of 72.15 (Dobrovolny et al., 2023).

Another study assesses the performance of two deep learning models, U-Net and Mask-RCNN, in the segmentation of human sperm components, including the head, acrosome, and nucleus. By implementing strategies such as data augmentation, cross-validation, hyperparameter tuning, and particularly transfer learning, significant improvements were achieved in the segmentation accuracy. The U-Net model, enhanced with transfer learning, demonstrated up to 95% overlap with manually segmented masks, as evaluated by the Dice coefficient, outperforming existing methods in sperm part segmentation. These findings highlight the impact of advanced deep learning techniques and transfer learning in pushing the boundaries of accuracy and reliability in sperm morphology analysis (Marín and Chang, 2021).

This paper is structured as follows: Section 2 reviews the current methodologies and their limitations, Section 3 describes the modified U-Net architecture and our approach to data augmentation, and subsequent sections discuss the implementation details, results, and clinical implications of our findings. Through this research, we contribute to the ongoing efforts to apply deep learning techniques in medical imaging, specifically in the enhancement of reproductive health diagnostics.

## 3 Methods

### 3.1 Data augmentation

Data augmentation plays a pivotal role in the training of deep learning models, especially in fields where the acquisition of large and varied datasets is challenging, such as in the segmentation of sperm cells. By artificially increasing the diversity of our training set, we aim to improve the model’s generalizability and robustness to variations in new, unseen images. This is particularly crucial in sperm segmentation, where variations in shape, size, orientation, and contrast of sperm cells across different samples can significantly affect model performance.

#### 3.1.1 Rotation

##### 3.1.1.1 Purpose

Sperm cells can be oriented in any direction in microscopic images. Introducing rotational variance through random rotations ensures our model does not learn orientation-specific features, thereby enhancing its ability to recognize sperm cells regardless of their angular position.

#### 3.1.2 Flip (horizontal and vertical)

##### 3.1.2.1 Purpose

Similar to rotations, horizontal and vertical flips augment the dataset with mirrored images, further training the model to be invariant to the orientation of sperm cells. This technique mimics the random orientation sperm cells can have on a slide, ensuring robust detection capabilities.

#### 3.1.3 Brightness and contrast adjustment

##### 3.1.3.1 Purpose

Variations in lighting and image quality are common in microscopic images due to differences in sample preparation and imaging equipment. Adjusting brightness and contrast simulates these variations, preparing the model to perform well under various imaging conditions.

#### 3.1.4 Gaussian noise

#### 3.1.5 Purpose

Microscopic images often contain noise introduced during the imaging process. By adding Gaussian noise, we simulate these imperfections, training our model to distinguish between noise and crucial features of sperm cells, enhancing its accuracy in real-world scenarios.

#### 3.1.6 Color augmentation

##### 3.1.6.1 Purpose

Although sperm cells are typically imaged in grayscale, variations in staining and imaging techniques can introduce subtle color variations. Color augmentation trains the model to be resilient to these variations, potentially useful in scenarios where color images are used or when slight color variations convey important information.

#### 3.1.7 Impact on model performance

The application of these augmentation techniques has had a noticeable impact on the performance of our deep learning models. Specifically, models trained on the augmented dataset demonstrated improved accuracy and generalizability when tested on unseen images. This is evidenced by a reduction in overfitting, as the model learns to focus on invariant features of sperm cells rather than memorizing specific images. Furthermore, the improved robustness to variations in sperm cell appearance and imaging conditions directly addresses the challenge of accurately segmenting closely adjacent sperm cells—a significant hurdle in automating sperm analysis.

By documenting the specific augmentation techniques and their motivations, we aim to provide a clear pathway for replicating our results and further advancing the field of automated sperm analysis. The detailed parameters and their purposes underline our methodical approach to overcoming dataset limitations and enhancing model performance, crucial steps toward the development of reliable computer-aided sperm analysis tools.

### 3.2 Methodological overview

In the pursuit of advancing automated sperm analysis, our research employs a multifaceted methodological approach grounded in deep learning and image processing technologies. Central to our investigation is the deployment of a modified U-Net architecture, chosen for its proven efficacy in medical image segmentation tasks. This choice is predicated on the architecture’s remarkable ability to accurately delineate intricate structures within complex biological images, a capability crucial for the segmentation of sperm cells from diverse and often challenging backgrounds. By adapting and refining this architecture, our study aims to address the critical gap in accurate, automated sperm detection and segmentation—an essential step forward in diagnosing and understanding male infertility.

The methodologies section that follows is designed to detail the systematic steps taken to tailor the U-Net architecture to the specific challenges inherent in sperm segmentation. Given the variable nature of sperm morphology and the presence of closely adjacent sperm cells in microscopy images, our adaptation focuses on enhancing the model’s precision and reliability under these conditions. Additionally, we incorporate comprehensive data augmentation strategies to ensure robust model training, mitigating the limitations imposed by the scarcity of labeled data in this domain. The rationale behind these methodological choices is twofold: to push the boundaries of current sperm segmentation capabilities and to establish a framework for future research to build upon. By elucidating these approaches, we invite readers to grasp the foundational strategies that guide our work, setting the stage for the technical intricacies that follow.

### 3.3 U-Net

In this paper we use UNet architecture which was first introduced by Ronneberger et al. (2015). This architecture was developed for Bio Medical Image Segmentation. UNet has two paths called encoder and decoder.

The main purpose of first path that also called contraction path is to extract feature maps and take the image information. The contraction path uses regular CNN architecture and contains of convolution and max pooling layers.

The decoder path which represents the uniqueness of UNet, combines the feature and spatial information and is used for localization (Sultana et al., 2020). This path which is also called symmetric expanding path, needs to accept image of any size. So, it does not contain any dense layer and only contains convolutional layers.

### 3.4 Other architectures

#### 3.4.1 LinkNet

Many architectures which are employed for segmentation, consist of several downsampling operations. This may lead to losing spatial information. Recovering this lost information is a challenging task due to the structure of most architectures. Because they mostly use only the downsampled output of encoder. Accordingly, LinkNet architecture proposes a way for linking each encoder and decoder (Chaurasia and Culurciello, 2017). This architecture attempts to recover spatial information by connecting input of each encoder to its corresponding decoder. So, the information would be recovered by upsampling operations in decoder. This novelty, allows the decoder to reduce its parameters which leads to more efficiency.

#### 3.4.2 MANet

This architecture proposed by Xu et al. (2021) for classification of COVID-19 positive cases from CXR images. The attention mechanisms can increase the abilities of a network and improve its functioning capabilities.

The attention block guides the model to concentrate on important parts of the input image. In other words, inappropriate features cannot affect the training process. This capability of model is essential in the analysis of medical pictures.

#### 3.4.3 U-Net++

U-Net++ is an encoder decoder architecture which has some advantaged over common Unet (Zhou et al., 2018).

The construction of U-Net mostly leads to losing details. As a result, most variants consist of some skip pathways between encoder and decoder. Similarly, in U-Net++, the feature maps of the encoder go through a dense convolution block. This can fill the space between the feature maps of the encoder and decoder. Another aspect of the proposed architecture is deep supervision which enables the model to perform on accurate or fast mode due to the situation.

#### 3.4.4 PSPNet

Pyramid scene parsing network was first introduced in ImageNet scene parsing challenge 2016 (Zhao et al., 2017). In the backbone of this architecture, the common convolutional layers are removed and dilated convolutional layers are used in order to improving the respective field.

The main capability of this architecture is to capturing the global context of the image. Conversely, fully convolutional networks are not able to classify pixels based on the context of the entire image. The pyramid pooling model is responsible for this capacity of PSPnet. The model is able to pool the feature map on a variety of scales. This approach helps the model to capture features in different resolution.

#### 3.4.5 FPN

As mentioned before, feature pyramids own the ability to distinguish objects at various scales. However, they are computationally expensive. The feature pyramid network has some advantages over pyramidal feature hierarchy without a noticeable cost (Lin et al., 2017). This architecture consists of two parts; The bottom-up pathway is the feedforward computation of the backbone ConvNet and the top-down pathway to up sampling higher resolution features. These two parts are connected to each other via lateral connections.

These models are compared in Table 1.

**Table 1:** Comparison of six different architectures that were used in this research.

Aspect	PSPNet	U-Net	U-Net++	MANet	LinkNet	FPN
Architecture type	Encoder-decoder	Encoder-decoder	Encoder-decoder	Encoder-decoder	Encoder-decoder	Backbone + top-down
Contextual information	Global	Local	Global	Global and local	Local	Multi-scale
Feature reuse	✓	✓	✓	✓	✓	✓
Skip connections	✓	✓	✓	✓	✓	✓
Upsampling technique	Interpolation	Transposed convolution	Concatenated convolution	Deformable convolution	Transposed convolution	Interpolation

According to the table, PSPNet and U-Net++ utilize global contextual information, while U-Net and LinkNet focus on local context. MANet combines both, and FPN leverages multi-scale context. There is a variety in upsampling techniques used, such as interpolation in PSPNet and FPN, transposed convolutions in U-Net and LinkNet, concatenated convolutions in U-Net++, and deformable convolutions in MANet.

The selection of deep learning models for this study was guided by a strategic evaluation of their architectural features, adaptability, and proven success in tasks akin to sperm segmentation. U-Net and its derivatives were particularly chosen for their exceptional performance in medical image analysis, characterized by their ability to capture fine-grained details and variations in complex biological structures. This is crucial for accurately segmenting sperm cells, which exhibit significant variability in shape, size, and texture. Furthermore, the architecture’s efficient use of convolutional networks to process and analyze image data makes it well-suited for handling the intricate patterns observed in sperm microscopy images. The decision to leverage these models is rooted in their potential to overcome the specific challenges of sperm analysis, such as distinguishing closely adjacent cells and dealing with the high dimensionality of image data. By harnessing the strengths of these architectures, we aim to push the boundaries of automated sperm segmentation, enhancing both the accuracy and reliability of infertility diagnostics.

Incorporating these models into our research framework also allows for an exploration of novel methodological enhancements and the application of transfer learning principles. This approach not only seeks to improve segmentation outcomes but also contributes to the broader scientific dialogue on refining deep learning techniques for biomedical imaging. The chosen models stand at the intersection of innovation and practicality, offering a robust foundation for addressing the nuanced requirements of sperm segmentation and setting a precedent for future advancements in the field.

### 3.5 Pseudocode

**Figure 1:**
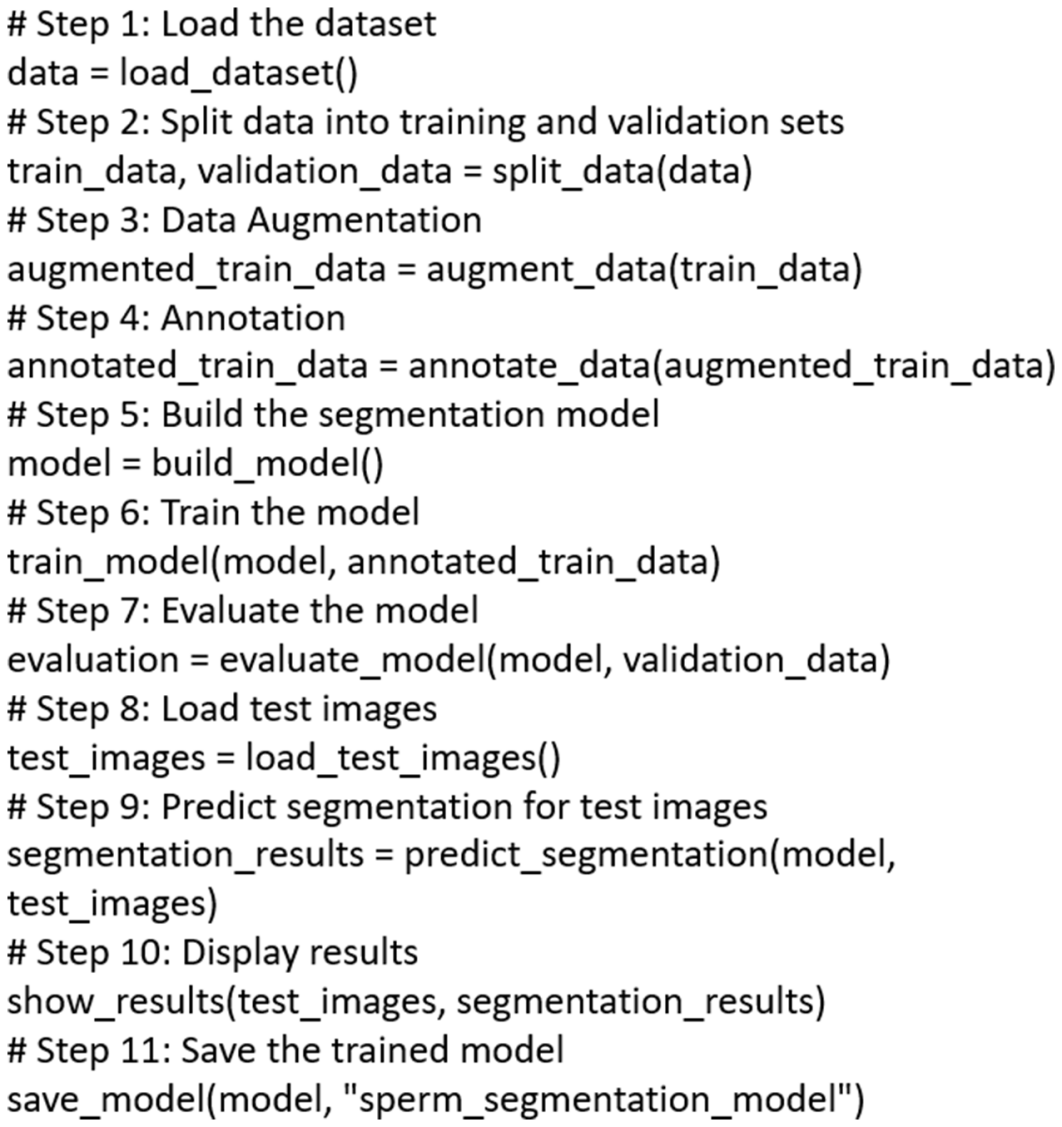
The pseudocode which outlines comprehensive sperm segmentation pipeline.

In our research, we meticulously followed a well-structured pseudocode to ensure a systematic and reproducible approach to sperm segmentation. This pseudocode encapsulates the key steps in our methodology. Firstly, we load and preprocess the dataset, splitting it into training and validation sets for robust model training. Data augmentation techniques are then applied to enhance the dataset’s diversity. Following this, we meticulously annotated the augmented data to create a comprehensive training set. Our next step involves constructing a sophisticated segmentation model designed specifically for sperm analysis. We then train this model using the annotated data. Subsequently, we evaluate the model’s performance using a validation dataset. For real-world applicability, we load test images, predict segmentations, and visually present the results. Finally, to preserve the model’s capabilities, we save it for potential future applications. This pseudocode serves as a structured blueprint, ensuring the transparency and reproducibility of our methodology in the field of sperm segmentation. The whole process is described in Figure 1.

## 4 Results

### 4.1 Dataset

We utilized the VISEM dataset, a comprehensive resource comprising videos and numerical data collected from 85 participants spanning an 18-year period. The videos exhibit a resolution of 640 × 480 pixels and vary in duration from 2 to 7 min. The dataset provides valuable insights into several critical aspects of sperm analysis, including sperm motility, sperm head concentration, total sperm headcount, ejaculate volume, sperm morphology, and viability.

The VISEM dataset is novel in two significant ways. Firstly, it is a multi-modal dataset containing diverse data sources such as videos, biological analysis data, and participant information. Secondly, it is the first dataset of its kind in the field of human reproduction, offering a unique and comprehensive resource for researchers. The data was originally collected to study the relationship between overweight, obesity, and male reproductive function, involving males aged 18 years or older. Participants were recruited between 2008 and 2013 from various sources, including the normal population, obesity clinics, and fertility clinics. The study was approved by the Regional Committee for Medical and Health Research Ethics, South East, Norway, and all participants provided written informed consent.

Each participant provided semen samples analyzed according to WHO recommendations, including assessments of sperm motility, concentration, total sperm count, ejaculate volume, morphology, and vitality. For video recording, a sample was placed on a heated microscope stage at 37°C and examined under 400x magnification using an Olympus CX31 microscope. Videos were captured using a UEye UI-2210C camera from IDS Imaging Development Systems in Germany and saved as AVI files. The resolution of the videos is 640 × 480 pixels with a frame rate of 50 frames per second.

The dataset also includes sperm fatty acid profiles, fatty acid compositions of serum phospholipids, demographic data, and WHO analysis data. It comprises over 35 gigabytes of videos and six CSV files (five for data and one mapping videos to participant IDs), along with a description file. Each video file is named with an ID, capture date, optional description, and the code of the person who assessed the video using WHO standards.

In the context of this research, we extracted a total of 100 frames from the original video recordings. Subsequently, each frame underwent meticulous annotation by an expert, distinguishing regions as either sperm or non-sperm. These annotated frames, along with their corresponding masks, are now publicly accessible via the following link: https://github.com/HananSaadat/Sperm-Segmentation-from-VISEM.

Researchers and scientists can leverage this dataset to advance their investigations in the field of sperm segmentation, object detection, and computer vision. We encourage its use for various research purposes and welcome contributions from the scientific community to further enrich this resource.

### 4.2 Baseline models

For this experiment, we compared the original Unet with 5 other architectures. The reason of using U-Net is due to its performance in the medical image segmentation. We also use other similar models in order to improve the results. These models are described in sec 2.

### 4.3 Metrics

**Figure 2:**
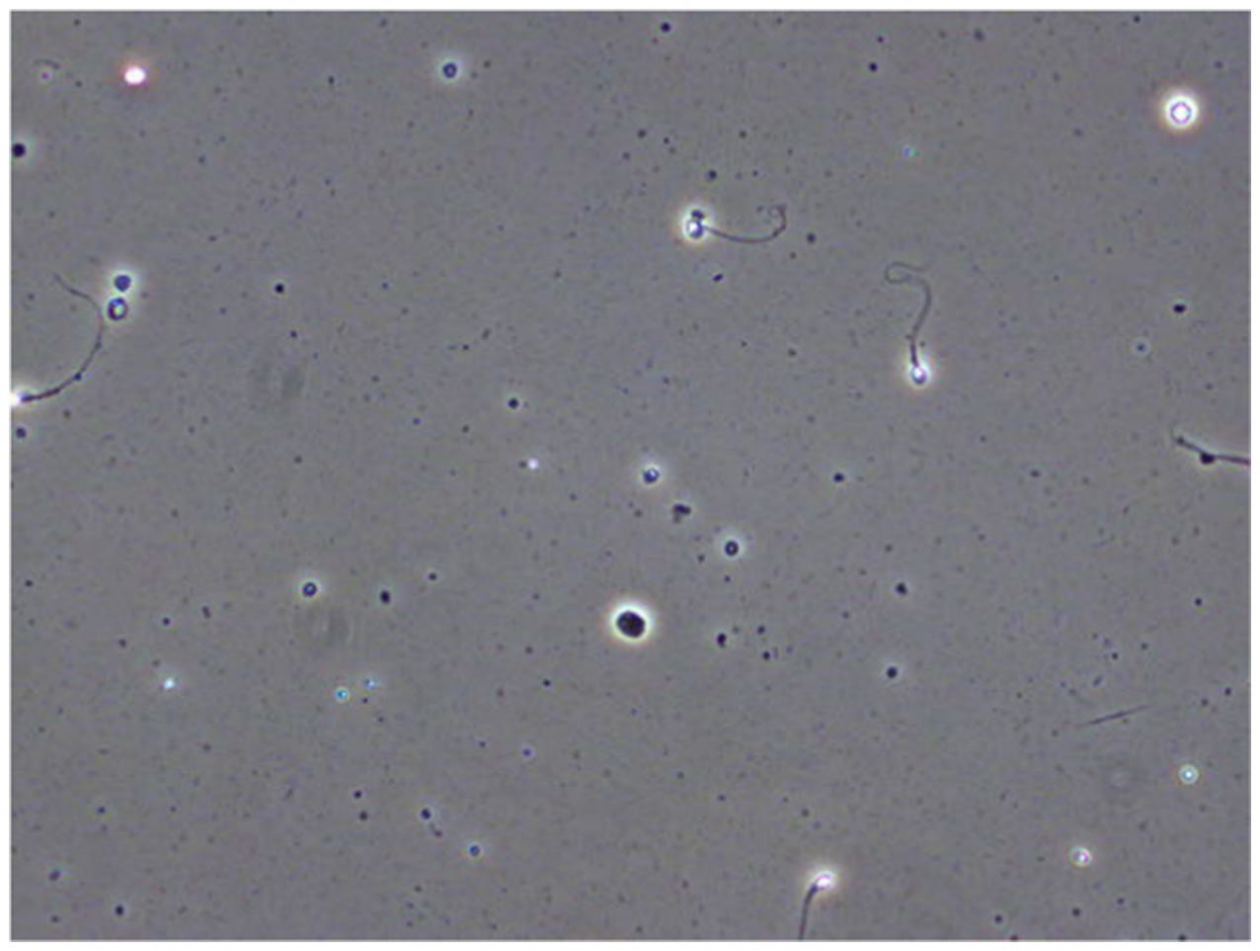
An example frame VISEM dataset. In all the frames most of the pixels are covered by non-sperms.

Pixel accuracy is a simple and easy metric to evaluate segmentation performance. It is the percent of pixels that classified correctly. However, due to the class imbalance in the images, as it is shown in Figure 2, one class dominates the image. So, even the worst models may have high accuracy and this metric is not a good choice for evaluating performance.

One of the well-known metrics for measuring the performance of medical image segmentation, are F-measure based metrics. In the segmentation of medical images, classes are mostly imbalanced and false positives matters. So, by combining the sensitivity and precision, these measures calculate the overlap between predicted segmentation and ground truth.

Two of the popular F-measure based are as follows:

The Intersection over Union (IoU): This metric, also known as Jaccard index is calculated by dividing the overlap between the predicted and ground truth annotation by the union of these.

**Figure 3:**
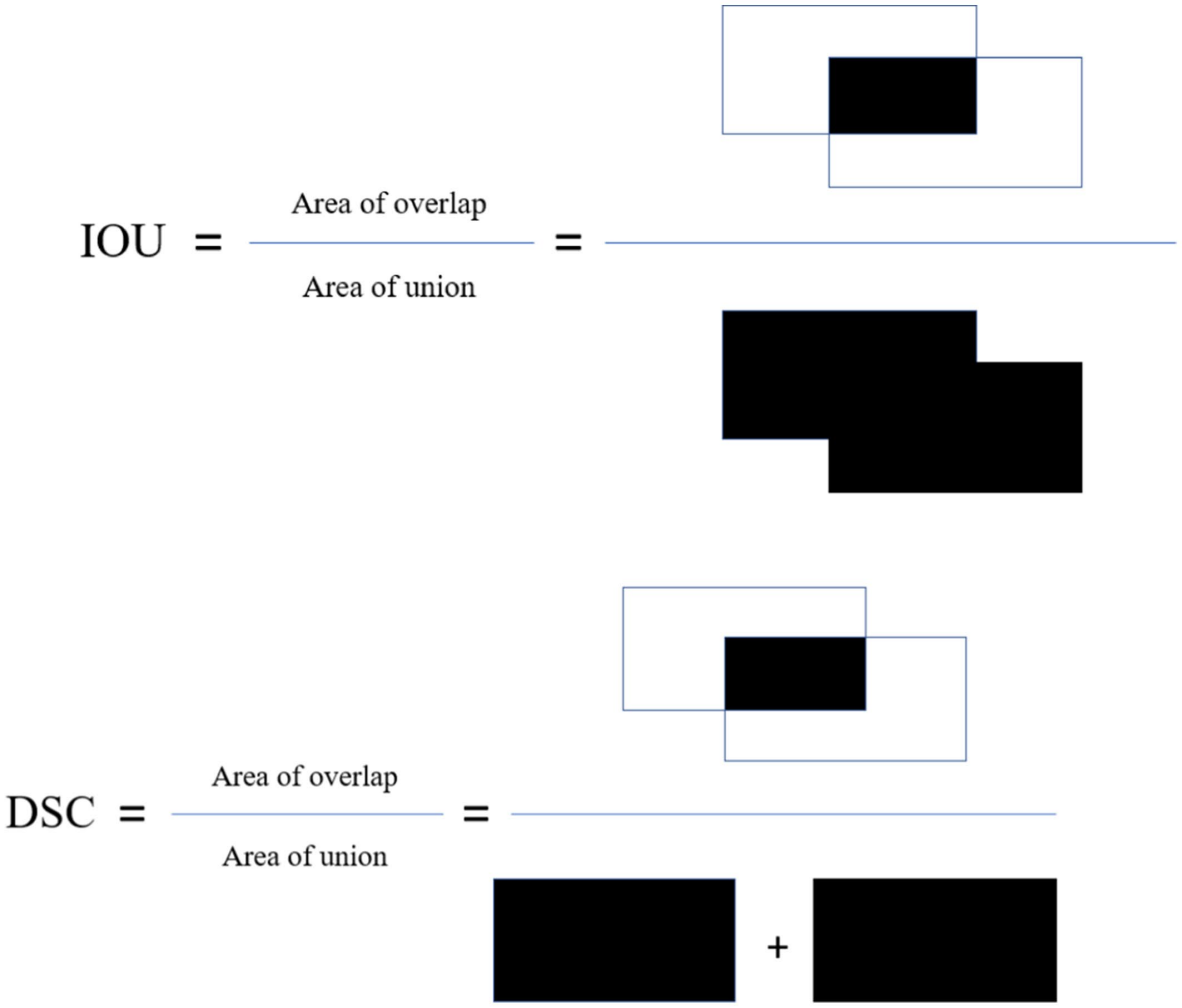
Visual explanation of dice and IOU.

Dice similarity coefficient (DSC): This metric is the most used metric in the medical image segmentation evaluation and its positively correlated with IoU. These metrics are described in Figure 3.


$$\text{Accuracy}=\frac{TP+TN}{TP+TN+FP+FN\text{.}}$$

$$\text{Dice}\;\text{Coefficient}=\frac{2\;x\;TP}{2\;x\;TP+FP+FN}$$

$$\text{Jaccard}\;\text{Index}=\;\frac{TP}{\;TP+FP+FN}$$

The performance of all architectures in the task of sperm segmentation is shown in Table 2. According to the table, choosing the most proper model is not simple. Each model is superior to others in some criteria. This choice depends on the problem and its challenges. Recall metric indicates how well the model was able to find sperms. In fact, the better this criterion is, the fewer sperms have been detected as something other than sperm. Precision metric indicates how well the model was able to distinguish non-sperm cells. The higher this criterion is, the less non-sperm cells are detected as sperm.

**Table 2:** Comparison of different architectures.

Model metric	Unet_Resnet18	UnetPlusPlus_Resnet18	FPN_Resnet18	Linknet_Resnet18	Manet_Resnet18	PSPNet_Resnet18	UnetPP_Resnet34	UnetPP_Efficient-b0
Jaccard	0.712617	0.372147	0.627135	0.661545	0.637536	0.452195	0.721984	0.671776
Dice	0.915384	0.767469	0.884476	0.897238	0.888411	0.809788	0.918576	0.900961
Acc	0.997167	0.985259	0.996244	0.996384	0.99637	0.993657	0.997231	0.996539
Precision	0.827058	0.380877	0.789677	0.755076	0.797224	0.621564	0.820875	0.766913
Recall	0.837399	0.941979	0.752892	0.842287	0.760926	0.623989	0.857001	0.844122

As mentioned before, the main challenge in the treatment process is that the non-sperm cells are recognized as sperm which makes precision metric the critical criteria.

In our study, the ROC (Receiver Operating Characteristic) curve emerges as a pivotal instrument for appraising the efficacy of our sperm segmentation model tailored for male infertility diagnosis. In the realm of binary classification, where distinguishing sperm from non-sperm cells is paramount, the ROC curve assumes a profound significance. It functions as a visual representation of the delicate balance between sensitivity (the model’s capacity to correctly identify sperm or true positives) and specificity (its aptitude for correctly discerning non-sperm cells or true negatives). By plotting the true positive rate (sensitivity) against the false positive rate (1-specificity), the ROC curve paints a dynamic picture of the model’s performance as we adjust the classification threshold. This flexibility allows us to fine-tune the threshold to meet the specific diagnostic criteria. Complementing the ROC curve is the Area Under the Curve (AUC), a single metric that quantifies the model’s overall performance. A higher AUC suggests a superior equilibrium between sensitivity and specificity, crucial for accurate sperm analysis.

By integrating the ROC curve into our research, we exemplify a robust methodology for evaluating our sperm segmentation model’s effectiveness. This visual representation not only underscores our comprehension of the nuanced trade-offs between sensitivity and specificity in a medical context but also underscores our dedication to optimizing the classification threshold to align with diagnostic objectives. This comprehensive analysis enhances the credibility of our study, delivering valuable insights into our model’s performance and its potential to influence male infertility diagnosis positively.

**Figure 4:**
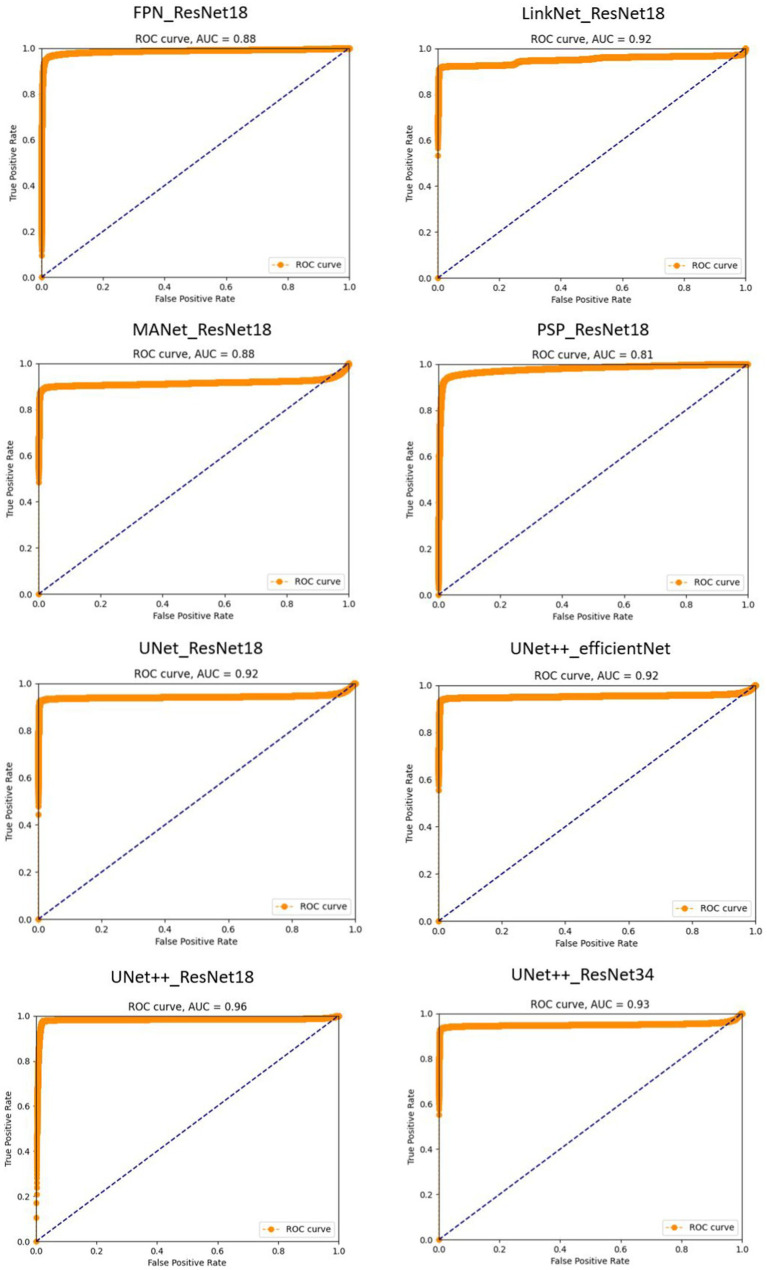
Receiver operating characteristic (ROC) curves for sperm segmentation models. The proximity of each curve to the upper-left corner of the plot reflects the model’s effectiveness, with curves closer to this corner indicating superior performance.

An ROC curve closer to the upper-left corner signifies superior performance, indicating that the model excels in identifying true positives while minimizing false positives. This dynamic visualization allows us to select the optimal threshold tailored to the unique diagnostic needs of sperm analysis. Furthermore, the Area Under the ROC Curve (AUC) quantifies the overall performance, providing a single metric to gauge the model’s accuracy in distinguishing sperm from non-sperm cells. These details are presented in Figure 4.

PSPNet with ResNet18 exhibits a lower AUC of 0.81. This indicates that it does not performs well in distinguishing sperm from non-sperm cells and there is room for improvement. The model’s sensitivity and specificity balance are good, but it may have a slightly higher false positive rate or lower true positive rate compared to the highest-performing models.

UNet++ with ResNet18 achieves an impressive AUC of 0.96 demonstrates its overall ability to discriminate between these classes, the precision score of 0.38 implies that when the model predicts a region as containing sperm, it is accurate only 38% of the time, leading to a relatively high rate of false positive identifications. Conversely, the recall score of 0.94 suggests that the model effectively identifies a substantial portion of the true sperm regions but may overlook some, potentially resulting in false negatives. This imbalance between precision and recall underscores the need for fine-tuning and threshold adjustments to achieve a more balanced performance, particularly concerning the critical task of distinguishing sperm from non-sperm regions.

UNet++ with ResNet34 on the other hand, excels in its performance with the highest AUC value. This suggests that this model is exceptionally effective in distinguishing sperm cells accurately from non-sperm cells. It strikes an impressive balance between high sensitivity and specificity, resulting in minimal false positives and false negatives.

**Figure 5:**
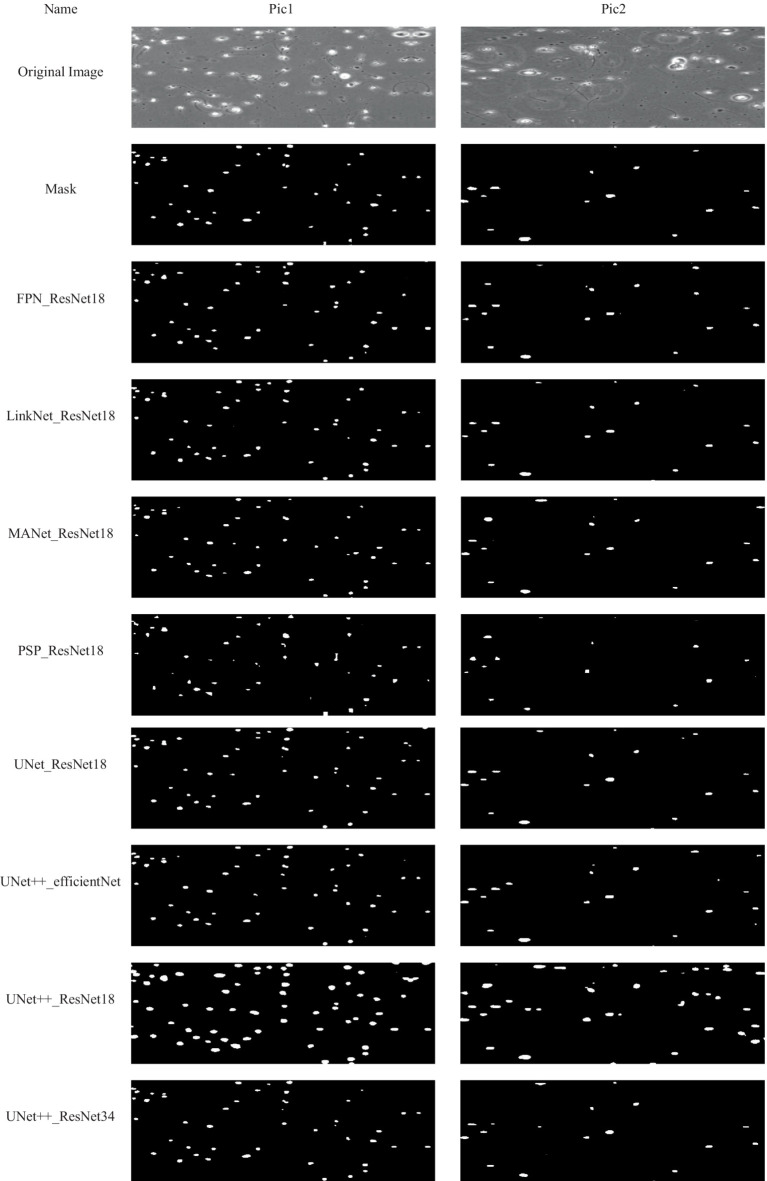
Examples of some results obtained by using the proposed method. Each column presents an image and corresponded results using the proposed method.

Some examples of performance of the model on sample images from test set, has been provided in Figure 5.

Of course, the model errors that can be seen in precision criteria and are related to recognizing non-sperm as sperm do not necessarily mean misdiagnosing non-sperm cells. Examining the output images of the model helps in better diagnosis. According to these images, the major part of the model’s error is in the part of the model where two sperms are located near each other. The model considers those two sperm as one sperm.

Considering that precision criteria is critical in the selection, U-Net with ResNet18 and U-Net++ with ResNet34 are proper models and their performance can be seen in the sample pictures. U-Net++ with ResNet18, despite its high accuracy, mistakenly recognized many pixels as sperm, and for this reason, recorded a high recall. But in the rest of the criteria, it performs bad.

## 5 Discussion

Various experimental approaches for detection and segmentation of sperm head are compared in this research. All the introduced models are the well-known methods for the segmentation of medical images.

Our study utilized the VISEM dataset, a valuable resource comprising videos and numerical data collected from 85 participants. These videos, with a resolution of 640 × 480 pixels and varying durations, offer a diverse and challenging dataset for sperm segmentation. The dataset includes information related to sperm motility, sperm head concentration, total sperm headcount, ejaculate volume, sperm morphology, and viability. These attributes provide a holistic view of the dataset’s potential applications in the field of reproductive biology and male infertility diagnosis.

To prepare the dataset for analysis, we extracted frames from the videos, and each frame was meticulously labeled as either sperm or non-sperm by an expert. This labeling process is crucial as it serves as the ground truth against which the performance of our segmentation models is evaluated.

In our experimentation, we compared the performance of the original U-Net architecture with five alternative models. The choice of U-Net as our baseline model was informed by its demonstrated efficacy in medical image segmentation tasks. Additionally, we incorporated other similar models to explore opportunities for performance improvement.

Our comprehensive evaluation, as presented in Table 1, revealed that each model exhibited strengths and weaknesses across various performance criteria. Selecting the most appropriate model for sperm segmentation is not a straightforward task, as each model excelled in specific aspects. The choice of model should be made with careful consideration of the specific problem and its associated challenges.

Our choice of evaluation metrics played a pivotal role in assessing the performance of the segmentation models. We acknowledge the limitations of using pixel accuracy, a simple yet potentially misleading metric, especially in the context of imbalanced datasets. The imbalance between sperm and non-sperm classes in the images can result in inflated accuracy scores, making it an unsuitable metric for performance evaluation.

To address this concern, we employed F-measure based metrics, including the Intersection over Union (IoU) and Dice similarity coefficient (DSC), which are well-established in the domain of medical image segmentation. These metrics account for both sensitivity and precision, offering a more balanced assessment of model performance.

Our study introduced the ROC curve as a powerful tool for evaluating the performance of our sperm segmentation models in the context of male infertility diagnosis. By illustrating the trade-offs between sensitivity and specificity, the ROC curve helps us fine-tune the classification threshold to meet specific diagnostic criteria. Additionally, the Area Under the ROC Curve (AUC) quantifies the model’s overall accuracy in distinguishing sperm from non-sperm cells.

Our results, as shown in Figure 4, highlight the varying performance of different models, with PSPNet exhibiting room for improvement and UNet++ with ResNet34 emerging as a top-performing model with a balanced sensitivity-specificity profile.

A critical challenge we identified in our study was the recognition of non-sperm cells as sperm, underscoring the importance of the precision metric. We conducted an in-depth error analysis, revealing that many errors occurred when two sperm cells were located in close proximity, leading the model to consider them as a single sperm.

To address this challenge, we recommend further investigations into model fine-tuning and threshold adjustments to achieve a more balanced performance, especially concerning the critical task of distinguishing sperm from non-sperm regions. Moreover, our findings highlight the need for domain-specific expertise in refining segmentation models.

## 6 Conclusion

In this study, we explored sperm segmentation’s critical role in male infertility diagnosis and reproductive biology research, utilizing the VISEM dataset. We aimed to advance understanding and model performance in this domain. The dataset’s rich attributes offered valuable avenues for male fertility assessment. Expertly labeled annotations laid the foundation for our analysis. Our evaluation of baseline models, including U-Net, revealed the challenge of selecting the optimal model. Each had distinct strengths and weaknesses, emphasizing the complexity of the task.

Acknowledging the limitations of pixel accuracy, we employed F-measure based metrics like IoU and DSC, offering a more balanced view of model performance. We introduced ROC analysis, showcasing sensitivity-specificity trade-offs. AUC quantified model accuracy, aiding sperm identification. Error analysis highlighted the challenge of non-sperm recognition, emphasizing precision’s importance.

In conclusion, our research advances sperm segmentation. By leveraging diverse datasets, novel metrics, and ROC analysis, we contribute to reproductive biology and male infertility diagnosis. Future work should focus on model refinement, threshold adjustments, and domain expertise integration to improve precision. This study has the potential to enhance healthcare practices and contribute to a broader understanding of reproductive biology.

## Data availability statement

The raw data supporting the conclusions of this article will be made available by the authors, without undue reservation.

## Author contributions

HS: Writing – original draft. MS: Writing – review & editing. M-RB: Conceptualization, Methodology, Writing – review & editing. BM: Writing – review & editing.
